# Pediatric *Mycobacterium bovis* Infection: Clinical Spectrum, BCG-Related Disease, Primary Immunodeficiency, and a Comprehensive Review of the Literature

**DOI:** 10.3390/vaccines14100867

**Published:** 2026-10-01

**Authors:** Neslihan Mete Atasever, Elif Dede, Kaya Köksalan, Ayper Somer, Selda Hançerli Törün

**Affiliations:** 1 Department of Pediatric Infection Diseases, Istanbul University Faculty of Medicine, Capa, Fatih, Istanbul 34093, Turkey; elifded_@hotmail.com (E.D.); ayper.somer@gmail.com (A.S.); selda.hancerli@istanbul.edu.tr (S.H.T.); 2 Department of Medical Microbiology, Istanbul University Faculty of Medicine, Çapa, Fatih, Istanbul 34093, Turkey; okoksalan@istanbul.edu.tr

**Keywords:** *Mycobacterium bovis*, Bacillus Calmette–Guérin (BCG), primary immunodeficiency, IL12RB1 deficiency, Mendelian susceptibility to mycobacterial disease (MSMD), pediatric tuberculosis

## Abstract

**Objectives:*** Mycobacterium bovis (M. bovis)* infection in children is uncommon but may remain unrecognized because routine laboratory methods often fail to distinguish it from *Mycobacterium tuberculosis*. In addition, Bacillus Calmette–Guérin (BCG)-associated *M. bovis* infection may represent the first manifestation of an underlying primary immunodeficiency. This study aimed to describe the clinical spectrum, diagnostic features, treatment, and outcomes of pediatric *M. bovis* infection and to review the contemporary literature on BCG-associated disease and primary immunodeficiency. **Methods:** We retrospectively reviewed 388 pediatric patients diagnosed with tuberculosis at a tertiary referral center between 1998 and 2025 and identified three children with culture- or PCR-confirmed *M. bovis* infection. Clinical presentation, microbiological findings, immunological evaluation, treatment, and outcomes were analyzed. In addition, a narrative review of representative pediatric studies on *M. bovis* infection, BCG-associated disease, and Mendelian susceptibility to mycobacterial disease (MSMD) was performed. **Results:** Two BCG-vaccinated 2-year-old children presented with persistent BCG-related lymphadenitis and were subsequently diagnosed with genetically confirmed IL12RB1 deficiency after identification of *M. bovis* BCG. The third patient, a previously healthy 15-year-old girl, developed pulmonary and gastrointestinal tuberculosis caused by non-BCG *M. bovis*. Recognition of *M. bovis* facilitated the optimization of antimycobacterial therapy, including the discontinuation of pyrazinamide where appropriate. Treatment decisions were individualized according to disease extent and the underlying immune status. Adjunctive interferon-γ therapy was administered to both children with IL12RB1 deficiency. All three patients achieved favorable clinical outcomes. A review of the published literature demonstrated that disseminated BCG disease is strongly associated with defects of the IL-12/IFN-γ pathway and frequently represents the first manifestation of primary immunodeficiency. **Conclusions:** Pediatric *M. bovis* infection should be considered in children with persistent or disseminated disease following BCG vaccination or with unusual manifestations of tuberculosis. Early species-level microbiological identification is essential because it directly influences antimicrobial therapy, while BCG-associated *M. bovis* infection should prompt comprehensive immunological and genetic evaluation for underlying primary immunodeficiency. Integration of microbiological diagnosis with immunological assessment enables individualized management and may improve clinical outcomes.

## 1. Introduction

*Mycobacterium bovis (M. bovis)* is a zoonotic member of the *Mycobacterium tuberculosis (M. tuberculosis)* complex that primarily infects cattle but remains an important cause of human tuberculosis, particularly in regions where bovine tuberculosis is endemic and consumption of unpasteurized dairy products persists. Consequently, the epidemiology of pediatric *M. bovis* infection remains incompletely understood [1,2,3,4].

Although zoonotic tuberculosis accounts for only a small proportion of all tuberculosis cases worldwide, its true incidence is likely underestimated because routine diagnostic assays frequently identify isolates only as members of the *M. tuberculosis* complex without species-level differentiation. Consequently, infections caused by *M. bovis* may remain unrecognized, particularly in children, in whom microbiological confirmation is often difficult because of the paucibacillary nature of the disease. Improved laboratory capacity for species identification is therefore essential not only for individual patient management but also for epidemiological surveillance of zoonotic tuberculosis [1,2,3,4,5].

Unlike *M. tuberculosis*, *M. bovis* is intrinsically resistant to pyrazinamide, making accurate species identification essential for appropriate antimicrobial therapy. Failure to recognize *M. bovis* infection may lead to inappropriate treatment regimens and delayed clinical improvement [4,5,6]. In addition, children presenting with Bacillus Calmette–Guérin (BCG)-associated disease may harbor underlying immunodeficiency. Although primary immunodeficiencies, particularly defects of the IL-12/IFN-γ pathway, represent the most frequent cause, acquired immunodeficiency, including untreated HIV infection, has also been associated with severe BCG disease, especially before widespread prevention of mother-to-child HIV transmission [7,8].

Although BCG vaccine strains and zoonotic *M. bovis* belong to the same bacterial species, they represent distinct clinical entities. BCG-associated disease occurs following vaccination and should primarily raise suspicion for an underlying inborn error of immunity, particularly defects affecting the IL-12/IFN-γ pathway. In contrast, non-BCG *M. bovis* infection is typically acquired through zoonotic exposure, such as consumption of unpasteurized dairy products or contact with infected cattle, and requires careful epidemiological assessment. Distinguishing between these two entities has important implications for both clinical management and immunological evaluation.

Beyond foodborne transmission, pediatric *M. bovis* infection has gained increasing clinical importance because of its association with BCG vaccination in children with underlying inborn errors of immunity. Persistent regional lymphadenitis, recurrent BCGitis, osteomyelitis, or disseminated BCG disease should alert clinicians to the possibility of defects involving the IL-12/interferon-γ signaling pathway. IL12RB1 deficiency is the most frequently reported genetic cause of Mendelian susceptibility to mycobacterial disease and is strongly associated with severe BCG-related complications [9,10,11,12,13,14].

Although several pediatric case reports and small case series have been published, few studies have simultaneously addressed the clinical spectrum of pediatric *M. bovis* infection, the diagnostic implications of species-level microbiological identification, the relationship between BCG-associated disease and primary immunodeficiency, and current evidence guiding clinical management. Therefore, we report three microbiologically confirmed pediatric cases of *M. bovis* infection and provide a focused review of the contemporary literature, emphasizing diagnostic challenges, therapeutic implications, immunological evaluation, and practical management.

## 2. Methods

Clinical specimens were examined by auramine O staining and cultured using the BACTEC MGIT 960 system (Becton Dickinson, Sparks, MD, USA) and Löwenstein–Jensen medium. Molecular characterization of mycobacterial isolates was performed at the Tuberculosis Molecular Epidemiology Laboratory, Aziz Sancar Institute of Experimental Medicine, Istanbul University, using DNA fingerprinting by spoligotyping. Spoligotyping differentiates most *M. bovis* genotypes from *M. bovis* BCG strains; however, isolates belonging to the SB0120/ST482 genotype cannot be distinguished from *M. bovis* BCG by this method alone. In Cases 1 and 2, both isolates belonged to the SB0120/ST482 genotype. Therefore, the diagnosis of BCG-associated disease was established by integrating the microbiological findings with the clinical context (history of BCG vaccination, compatible clinical presentation, and the patients’ age). No additional confirmatory molecular assay was performed.

Disseminated BCG disease was defined as involvement of two or more non-contiguous anatomical sites, according to previously published criteria [7,15].

A narrative literature review was performed to provide contemporary evidence on pediatric *Mycobacterium bovis* infection and BCG-associated disease. PubMed/MEDLINE and Scopus were searched from database inception to February 2026 using combinations of the terms “*Mycobacterium bovis*”, “BCG”, “BCG disease”, “BCGitis”, “BCGosis”, “child”, “pediatric”, “primary immunodeficiency”, “IL12RB1 deficiency”, and “Mendelian susceptibility to mycobacterial disease”. Original articles, case reports, case series, and relevant review articles published in English were screened. Studies were selected according to their relevance to the clinical presentation, microbiological diagnosis, immunological evaluation, and management of pediatric *M. bovis* infection. The selected studies were chosen to illustrate the spectrum of previously reported pediatric cases and their relevance to the present series.

## 3. Case 1

A previously healthy 2-year-old boy who had received BCG vaccination at 2 months of age initially presented with left axillary lymphadenopathy, followed by bilateral cervical lymphadenopathy at 6 months of age. Acid-fast bacilli were identified in lymph node biopsy specimens. Because no clinical improvement was observed after 2 months of standard anti-tuberculosis treatment, further microbiological evaluation was performed. *M. bovis* BCG was isolated from gastric aspirate and lymph node tissue cultures, consistent with disseminated BCG disease. Chest imaging also demonstrated pulmonary involvement. Accordingly, Case 1 fulfilled the published definition of disseminated BCG disease because both lymphatic and pulmonary sites were involved. Because chronic granulomatous disease was initially suspected, adjunctive recombinant interferon-γ therapy was initiated while immunological investigations were ongoing. Subsequent genetic testing identified IL12RB1 deficiency. The patient’s clinical condition gradually improved during follow-up, and interferon-γ therapy is ongoing.

## 4. Case 2

A previously healthy 2-year-old girl who had received BCG vaccination at 2 months of age presented with persistent left axillary lymphadenopathy and recurrent oral candidiasis for approximately one year. Polymerase chain reaction (PCR) analysis of an excised lymph node was positive for *M. bovis* BCG. Anti-tuberculosis treatment was initiated, and fluconazole therapy was started for oral candidiasis. Because of persistent and recurrent lymphadenopathy, further immunological evaluation was performed and revealed IL12RB1 deficiency, the most common genetic defect associated with Mendelian susceptibility to mycobacterial disease. After identification of *M. bovis* BCG, pyrazinamide was discontinued and streptomycin was added to isoniazid, rifampicin, and ethambutol. The patient’s symptoms resolved, and adjunctive interferon-γ therapy was initiated because of IL12RB1 deficiency. She remains clinically stable during follow-up.

## 5. Case 3

A previously healthy 15-year-old girl with a history of BCG vaccination presented with one month of fatigue and one week of intermittent fever and chest pain. Imaging studies demonstrated bilateral pleural effusions measuring up to 50 mm and 65 mm of free intra-abdominal fluid. Because of these findings, an underlying malignancy was initially suspected but subsequently excluded. Acid-fast bacilli were identified in laparoscopic biopsy specimens, and culture yielded non-BCG *M. bovis*. Following microbiological identification, pyrazinamide was discontinued from the treatment regimen. Progressive clinical and laboratory improvement was observed during follow-up, and treatment is ongoing. Immunological evaluation revealed no evidence of an underlying primary immunodeficiency.

The demographic characteristics, clinical manifestations, microbiological findings, immunological evaluation, treatment, and outcomes of the three patients are summarized in Table 1.

**Figure 1:**
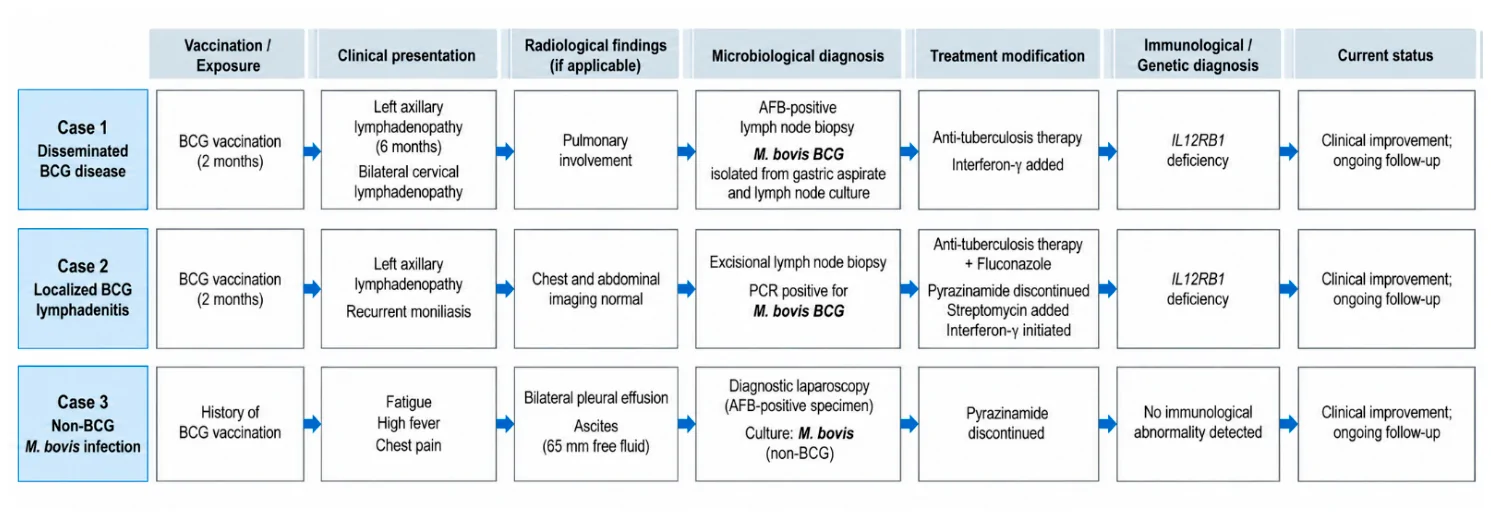
Clinical timelines of the three pediatric patients with *Mycobacterium bovis* infection. The figure summarizes vaccination or exposure history, clinical presentation, radiological findings, microbiological diagnosis, treatment modifications, immunological evaluation, and current clinical status. Abbreviations: AFB, acid-fast bacilli; BCG, Bacillus Calmette–Guérin; IL12RB1, inter-leukin-12 receptor β1; PCR, polymerase chain reaction.

The clinical course of all three patients is summarized in Figure 1.

## 6. Discussion

Our study expands the limited evidence on pediatric *M. bovis* infection by integrating three illustrative cases with a comprehensive review of the published literature. The present case series illustrates the heterogeneous clinical spectrum of pediatric *M. bovis* infection, ranging from localized BCG-associated disease to disseminated infection caused by underlying MSMD. The chronological clinical courses of the three patients are illustrated in Figure 1. Two of our patients had genetically confirmed IL12RB1 deficiency, underscoring the pivotal role of the IL-12/IFN-γ pathway in host defense against mycobacteria. Furthermore, microbiological identification of *M. bovis* enabled appropriate modification of antimicrobial therapy by recognizing the organism’s intrinsic resistance to pyrazinamide. Collectively, these findings emphasize the importance of early species identification, comprehensive immunological evaluation, and individualized management in children with suspected *M. bovis* infection.

Our findings are consistent with previously published pediatric studies. The largest pediatric cohort from Mexico demonstrated that more than half of children with culture-confirmed *M. bovis* infection had an underlying primary immunodeficiency, predominantly involving defects of the IL-12/IFN-γ axis, while extrapulmonary disease represented the most common clinical presentation. Similar observations have subsequently been reported in cohorts of children with BCG-associated disease and Mendelian susceptibility to mycobacterial disease, in which IL12RB1 deficiency emerged as one of the most frequent genetic causes of disseminated BCG infection [11,16,17,18]. Recent cohort studies have further confirmed that disseminated BCG disease is strongly associated with inborn errors of immunity and should prompt comprehensive immunological investigation. To better place our findings in the context of the existing evidence, key published studies on pediatric *M. bovis* infection, BCG-associated disease, and primary immunodeficiency are summarized in Table 2 [9,10,12,13,17,18,19].

The diagnosis of pediatric *M. bovis* infection remains challenging because routine microbiological investigations generally identify isolates only as members of the *M. tuberculosis* complex, and species-level identification is not routinely performed in many laboratories. Although microbiologically confirmed pediatric *M. bovis* infection appears to be uncommon, limited species-level testing may contribute to underrecognition in selected patients, particularly those with BCG-associated disease or relevant epidemiological exposures. Our findings further support incorporating species-level identification into the diagnostic work-up of children with persistent lymphadenitis, disseminated BCG infection, or atypical extrapulmonary tuberculosis whenever microbiological confirmation is available [12,20,21]. Exposure history remains an important component of the diagnostic evaluation. In countries where bovine tuberculosis remains endemic, including parts of Latin America, Africa, South Asia, and the Middle East, consumption of unpasteurized milk and dairy products from infected cattle continues to represent the principal route of zoonotic *M. bovis* transmission. In children, foodborne infection frequently presents with cervical lymphadenitis, abdominal lymphadenopathy, or gastrointestinal tuberculosis, whereas pulmonary disease is less common than in *M. tuberculosis* infection. Careful assessment of dietary exposure and epidemiological risk factors may therefore increase clinical suspicion and guide appropriate microbiological investigations [1,3,5].

Although BCG vaccination remains one of the most widely administered vaccines worldwide and provides effective protection against severe forms of childhood tuberculosis, serious BCG-related complications are rare and occur predominantly in children with underlying primary immunodeficiencies [10,12,14,18]. Disseminated BCG disease (BCGosis) is now recognized as one of the most important clinical indicators of defects affecting the IL-12/IFN-γ signaling pathway, particularly IL12RB1 deficiency [9,10,12,13,18]. In our series, two of the three children developed BCG-related disease and were subsequently diagnosed with genetically confirmed IL12RB1 deficiency, underscoring the importance of considering an underlying immunological disorder whenever persistent, recurrent, or disseminated disease develops after BCG vaccination. Although primary immunodeficiencies account for most contemporary pediatric cases of disseminated BCG disease, acquired immunodeficiency, particularly untreated HIV infection, has historically also been associated with severe BCG-related complications before the widespread implementation of prevention of mother-to-child HIV transmission programs [7,8]. These observations are consistent with recent cohort studies demonstrating that BCG-related complications frequently represent the first manifestation of MSMD [10,12,13,18]. Consequently, early recognition of atypical BCG disease should prompt comprehensive immunological and genetic evaluation, allowing earlier diagnosis and individualized treatment [11,12,13,18]. In the present series, Case 1 fulfilled the definition of disseminated BCG disease because it involved both lymphatic and pulmonary sites, whereas Case 2 was classified as localized BCG lymphadenitis.

The close association between disseminated BCG disease and inherited defects of the IL-12/IFN-γ pathway has been consistently demonstrated over the past two decades [9,11,12]. In our series, two of the three patients were diagnosed with genetically confirmed IL12RB1 deficiency, highlighting that BCG-related disease may represent the first clinical manifestation of an underlying inborn error of immunity. These findings support current recommendations that children with persistent, recurrent, or disseminated BCG disease should undergo comprehensive immunological evaluation, including genetic testing whenever available [9,10,11,12].

Accurate species identification has important therapeutic implications in pediatric mycobacterial infections [22,23]. Although *M. bovis* belongs to the *M. tuberculosis* complex, it is intrinsically resistant to pyrazinamide because of naturally occurring mutations in the *pncA* gene [23,24]. Consequently, failure to distinguish *M. bovis* from *M. tuberculosis* may result in inappropriate treatment regimens and unnecessary exposure to an ineffective drug [22,25]. In our patients, microbiological confirmation of *M. bovis* directly influenced treatment decisions by enabling discontinuation of pyrazinamide and optimization of the antimycobacterial regimen.

Appropriate antimicrobial management of *M. bovis* infection remains challenging, particularly in children with BCG-associated disease and underlying primary immunodeficiency. Once *M. bovis* is identified, pyrazinamide should be discontinued because all BCG vaccine strains are intrinsically resistant to this agent [26]. Current evidence suggests that treatment should be individualized according to disease severity, microbiological susceptibility, and the underlying immune defect. In patients with disseminated BCG disease, prolonged multidrug antimycobacterial therapy is frequently required, and second-line agents may be necessary in severe or refractory cases [26]. Adjunctive interferon-γ therapy has been successfully used in selected patients with IL12RB1 deficiency and other forms of MSMD and may improve clinical outcomes when combined with appropriate antimycobacterial therapy [11,26].

**Figure 2:**
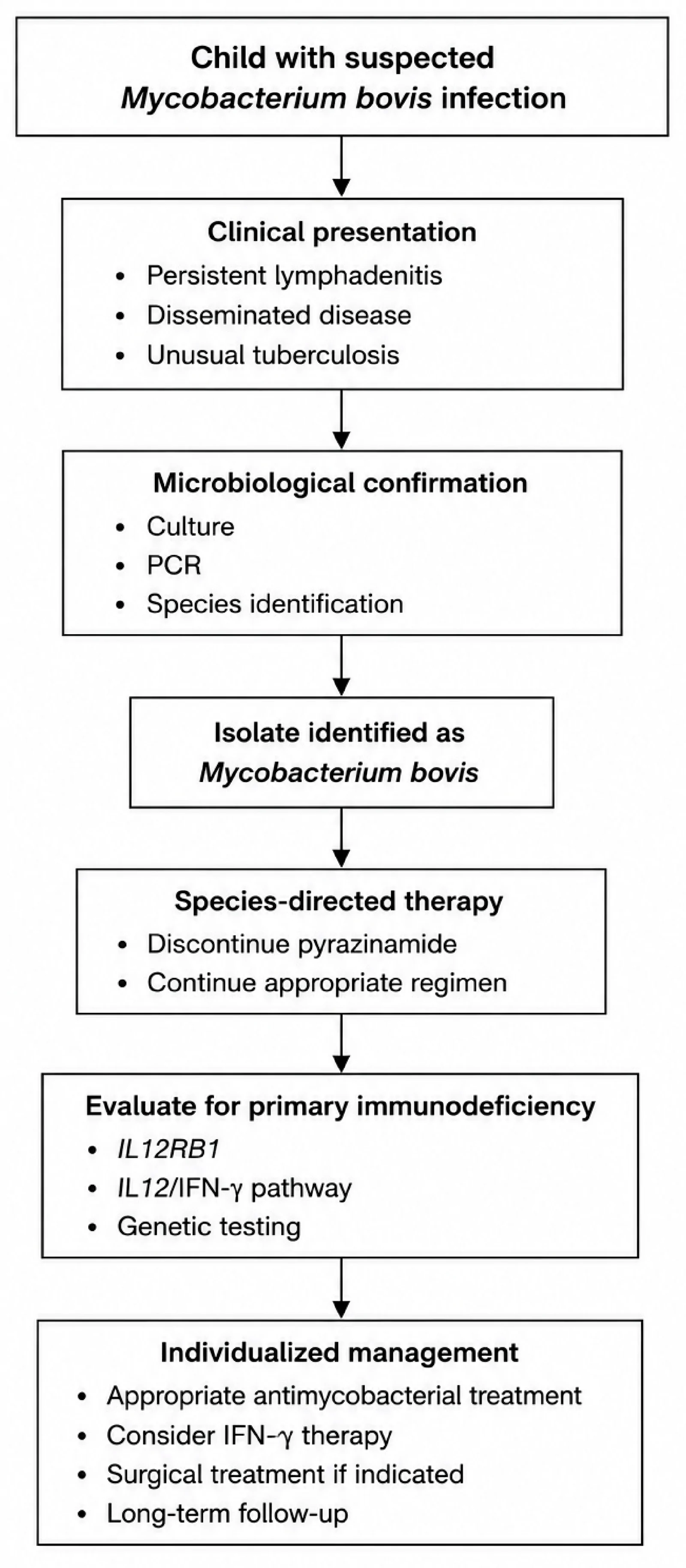
Proposed clinical approach to pediatric *Mycobacterium bovis* infection based on the present case series and the available literature.

Accurate species identification is also essential for optimal patient management. From a practical perspective, clinicians should be aware of the diagnostic limitations of commonly used tuberculosis tests. Neither the tuberculin skin test (TST) nor currently available interferon-γ release assays (IGRAs) can differentiate *M. bovis* from *M. tuberculosis*. Likewise, rapid molecular assays such as Xpert MTB/RIF Ultra detect organisms belonging to the *M. tuberculosis* complex without species-level identification. Definitive differentiation of *M. bovis* requires mycobacterial culture followed by molecular species identification, such as PCR-based assays or whole-genome sequencing. Therefore, species-level testing should be considered in children with BCG-associated disease, extrapulmonary tuberculosis, or epidemiological risk factors suggestive of zoonotic exposure [5,27,28]. These findings support a multidisciplinary approach involving pediatric infectious diseases, clinical microbiology, immunology, and genetics for the optimal management of children with suspected BCG-related disease. A proposed clinical approach based on the present cases and the available literature is illustrated in Figure 2.

Recognition of microbiologically confirmed pediatric *M. bovis* infection may contribute to surveillance of zoonotic tuberculosis and facilitate identification of potential transmission routes. From a clinical perspective, species-level identification facilitates optimization of antimicrobial therapy, while treatment should be individualized according to disease extent and the underlying immune status. In addition, identification of *M. bovis* may prompt evaluation for underlying inborn errors of immunity. Future multicenter collaborative studies are needed to better define the epidemiology and management of pediatric *M. bovis* infection [3,5,13,20,21,26].

Our study has several strengths. First, all cases had microbiologically confirmed *M. bovis* infection, allowing species-specific interpretation of clinical presentation and treatment. Second, comprehensive immunological and genetic evaluation identified IL12RB1 deficiency in two patients, providing a clear pathogenic explanation for disseminated BCG disease. Third, unlike most previously published reports, our findings are interpreted in the context of the available pediatric literature and directly compared with representative contemporary studies (Table 2), enabling a broader understanding of the clinical spectrum of pediatric *M. bovis* infection, BCG-associated disease, and primary immunodeficiency. Finally, we propose a practical diagnostic algorithm (Figure 2) integrating the clinical lessons derived from our cases with the current literature, which may facilitate earlier recognition and multidisciplinary management of children with suspected *M. bovis* infection.

Nevertheless, several limitations should be acknowledged. This was a retrospective case series from a single tertiary referral center, and the number of patients was inherently limited because of the rarity of pediatric *M. bovis* infection. In addition, the heterogeneity of clinical presentations and underlying immunological disorders limits direct comparison with previously published cohorts. Finally, because of the observational nature of this study, causal inferences regarding treatment efficacy cannot be established. Larger multicenter studies and international collaborative registries are needed to better define the epidemiology, optimal diagnostic approach, and management strategies for pediatric *M. bovis* infection [11,13,26].

## 7. Conclusions

Pediatric *Mycobacterium bovis* infection is uncommon but should be considered in children with persistent BCG-related disease, lymph node or extrapulmonary tuberculosis, or relevant epidemiological exposures. Species-level identification is important because it directly influences antimicrobial therapy and may uncover underlying immunodeficiency, particularly defects affecting the IL-12/IFN-γ pathway. Early recognition through multidisciplinary collaboration among pediatric infectious disease specialists, microbiologists, immunologists, and geneticists can facilitate appropriate treatment and long-term management.

## Figures and Tables

**Table 1:** Clinical characteristics, microbiological findings, immunological evaluation, and outcomes of the three pediatric *Mycobacterium bovis* cases.

Case	Age/Sex (Years)	Clinic Presentation	Isolate	Underlying Immunodeficiency	Diagnostic Method	Treatment Modification	Clinical Outcome
Case 1	2/M	Cervical and axillary lymphadenitis	BCG	IL12RB1 deficiency	Culture + PCR	PZA omitted + IFN-γ	Recovered
Case 2	2/F	Axillary lymphadenitis + recurrent oral candidiasis	BCG	IL12RB1 deficiency	PCR	PZA omitted + STR + IFN-γ	Recovered
Case 3	15/F	Pulmonary and gastrointestinal TB	Non-BCG	None	Culture + PCR	PZA omitted	Recovered

Abbreviations: BCG, Bacille Calmette–Guérin; F, female; IFN-γ, interferon-gamma; M, male; PCR, polymerase chain reaction; PZA, pyrazinamide; STR, streptomycin; TB, tuberculosis. BCG refers to the vaccine strain of *M. bovis*.

**Table 2:** Summary of key published studies on pediatric *Mycobacterium bovis* infection, BCG-associated disease, and primary immunodeficiency.

Study	Study Design/Population	Main Findings
Gonzalez Saldaña et al. [17], 2019	Retrospective cohort (22 children)	High prevalence of primary immunodeficiency, predominantly IL-12/IFN-γ pathway defects; extrapulmonary disease was the most frequent presentation.
Drysdale et al. [19], 2020	Case series (8 children)	Disseminated BCG disease occurred in a child with IL12RB1 deficiency; drug resistance emerged during prolonged therapy.
Azarsiz et al. [9], 2021	Cohort study (12 children with MSMD)	Six patients had IL12RB1 deficiency; BCGitis/BCGosis was the most frequent presentation; survival improved with prolonged antimycobacterial therapy and IFN-γ.
Soyak Aytekin et al. [10], 2021	Case series of children with BCGitis/BCGosis	IL12RB1 deficiency was the most common genetic diagnosis; disseminated disease predominated in affected children.
Xia et al. [13], 2022	National cohort (65 patients with MSMD)	BCG complications occurred in 92% of patients; IL12RB1 deficiency was among the most common genetic defects.
Fazlollahi et al. [12], 2024	Multicenter cohort (197 patients with IEI)	Systemic BCG disease was strongly associated with primary immunodeficiency; IL12RB1 deficiency represented one of the principal MSMD-associated defects.
Xiao Ma et al. [18], 2025	Single case report	Early molecular diagnosis and prolonged anti-BCG treatment with IFN-γ resulted in excellent clinical recovery.

Abbreviations: BCG, Bacillus Calmette–Guérin; IFN-γ, interferon-gamma; IL12RB1, interleukin-12 receptor β1 deficiency; IEI, inborn errors of immunity; MSMD, Mendelian susceptibility to mycobacterial disease. Only studies considered most relevant to pediatric *M. bovis* infection, BCG-associated disease, and primary immunodeficiency are summarized.

## Data Availability

The data presented in this study are available from the corresponding author upon reasonable request. The data are not publicly available due to privacy and ethical restrictions.
